# Attentional bias for trauma-related words: exaggerated emotional Stroop effect in Afghanistan and Iraq war veterans with PTSD

**DOI:** 10.1186/1471-244X-13-86

**Published:** 2013-03-14

**Authors:** Victoria Ashley, Nikki Honzel, Jary Larsen, Timothy Justus, Diane Swick

**Affiliations:** 1 Research Service, Veterans Affairs Northern California Health Care System, Martinez, CA, USA; 2 The Department of Neurology, University of California, Davis, CA, USA

**Keywords:** Posttraumatic stress disorder, PTSD, Stroop, Habituation, Trauma, Interference

## Abstract

**Background:**

Post-traumatic stress disorder (PTSD) involves debilitating symptoms that can disrupt cognitive functioning. The emotional Stroop has been commonly used to examine the impact of PTSD on attentional control, but no published study has yet used it with Afghanistan and Iraq war veterans, and only one previous study has compared groups on habituation to trauma-related words.

**Methods:**

We administered the emotional Stroop, the Beck Depression Inventory (BDI), and the PTSD Checklist (PCL) to 30 veterans with PTSD, 30 military controls, and 30 civilian controls. Stroop word types included Combat, Matched-neutral, Neutral, Positive and Negative.

**Results:**

Compared to controls, veterans with PTSD were disproportionately slower in responding to Combat words. They were also slower and less accurate overall, did not show interference on Negative or Positive words relative to Neutral, and showed a trend for delayed but successful habituation to Combat words. Higher PCL and BDI scores also correlated with larger interference effects.

**Conclusions:**

Because of its specificity in detecting attentional biases to trauma-related words, the emotional Stroop task may serve as a useful pre- and post task with intervention studies of PTSD patients.

## Background

Post-traumatic stress disorder (PTSD) involves long-term alteration of physiological and emotional functioning following exposure to horrific events, and typically involves intrusive cognitive and emotional phenomena such as nightmares, flashbacks, memory deficits and biases in attentional allocation [1]. The combat theaters of the Afghanistan and Iraq wars, Operation Enduring Freedom and Operation Iraqi Freedom (OEF/OIF), involved high rates of PTSD (estimated at 10 to 17%), multiple and extended troop deployments, high wound survivability rates and large numbers of traumatic brain injuries (TBI) [2,3]. Numerous studies have examined the cognitive and emotional impact of PTSD on returning US service members. However, no studies that we are aware of have used the emotional Stroop to assess this population, a task commonly used to examine attention biases in anxiety and depressive disorders, including PTSD [4-6], and particularly in war veterans with PTSD [7-13].

Individuals with PTSD have typically been exposed to extreme emotional stress resulting in altered physiology and disrupted cognitive functioning. Theories of cognitive function alterations in PTSD suggest that the fear network – a system allowing the rapid detection and response to threats – becomes dysregulated and hyperresponsive, leading to an attentional bias toward the detection of potential threats and an inability to disengage from perceived trauma-related reminders [14]. Consequently, the availability of cognitive resources to engage non-threat goals and task demands is decreased. Fear conditioning paradigms also reveal distinct alterations in PTSD patients, including the failure to consolidate and retrieve extinction learning, which is thought to play a role in the intractable nature of the trauma memory [14,15].

The emotional Stroop task, a variant of the classic Stroop task, can assess attentional biases in trauma-exposed individuals by comparing reaction time (RT) differences to name the font color of trauma-related words relative to neutral words, with instructions to ignore the meaning of the words. While healthy individuals are typically slower to name the colors of negative words [16], this effect is often more robust in individuals with PTSD when naming the colors of trauma-related words [5,6] (however, see [17]). The mechanisms of interference in the emotional Stroop task have been debated. Earlier PTSD studies, in the 1990’s, concluded that hypervigilance for trauma-related words facilitated or ‘attracted’ attention to the meaning of those words automatically, impacting the ability to name the word color [10,11,18]. Later studies found that, more than facilitation, the difficulty appeared to be with disengaging attention from trauma-related words [19-21]. Recent studies suggest roles for both facilitated attention and difficulty in disengagement [22]. In addition, attentional biases may be maintained by diverting attention sooner and longer with trauma-related reminders.

Importantly, the emotional Stroop can be used to examine key questions about cognitive alterations in PTSD, such as whether attentional biases are related to mere trauma exposure, or are specific to PTSD; whether the severity of PTSD symptoms correlates with emotional Stroop task performance; and if so, whether a PTSD patient can recover from exposure to trauma-related stimuli over the course of a block of emotional Stroop trials in order to return attention to task demands. Our study was designed to address these questions.

Whether emotional Stroop interference from trauma-related words reflects specific characteristics of PTSD, or only the consequences of exposure to traumatic events, has been debated in the literature. Some have argued that RT differences seen in an emotional Stroop may be due to a self-relevant event, the trauma, and not to PTSD [17]. And a recent study on visual attention to threatening stimuli using eye-tracking, found that Iraq veterans with PTSD were biased towards all negative valence stimuli, rather than just Iraq-specific stimuli [23]. Similarly, a meta-analysis suggests that the emotional Stroop task indexes exposure to trauma, rather than PTSD itself [5]. However, several other studies have found results supporting the idea that the emotional Stroop can index PTSD specifically [10,11,20,24,25]. In order to distinguish between trauma-exposed individuals with and without PTSD, we included a military control group (MC) in our study that had experienced the same trauma environment as PTs (19 out of 30 MCs were deployed to Iraq or Afghanistan during the OEF/OIF wars). Additionally, to reveal any possible effects broadly due to military work conditions and lifestyles, we also included a healthy civilian control group (CC).

Although PTSD patients often show significant interference to only trauma-related stimuli, rather than general negative or threat-related stimuli [10,11], other studies do not agree on this finding [5,9,17]. A 1996 study [9] found emotional Stroop interference effects for veterans color-naming high-threat words unrelated to their trauma, suggesting that PTSD patients may display interference effects from all high-threat words, rather than just trauma-related words. However, findings that PTSD patients show a specific bias for trauma-related words, and not generally negative or threatening words would support the idea that the emotional Stroop indexes PTSD, rather than mere exposure to trauma (with or without PTSD). In order to examine the apparent specificity in PTSD for threat-related words, rather than comparing only threat-related versus matched neutral words, we used five different word types in the current study: Combat, Matched-neutral, Negative, Positive and Neutral. Combat and Matched-neutral were each compared, and then separately, Negative, Positive and Neutral were each compared. This separation kept variables such as word frequency, valence, arousal, and other properties as consistent as possible across comparisons. Furthermore, many emotional Stroop studies of PTSD have included small numbers of words and have repeated them. However, when words are repeated, a potential confound is introduced between whether any observed habituation is due to perceiving the same word more than once, or to adapting to the semantic content of the word, or both. Consequently, we used all unique words in the current study.

The intractable nature of the fear response to trauma reminders in PTSD is often cited as a hallmark of the disorder, involving a unique difficulty for PTSD sufferers to habituate, or adapt to, such reminders. For example, some veterans with PTSD who participated in our study described experiencing overwhelming feelings of anger and fear upon getting caught in traffic jams, an experience that reminded them of their vulnerability to roadside explosive attacks in Iraq. Despite knowing that roadside bombings would be unlikely to occur in the US, the debilitating overwhelming emotional response was inevitable. Such an inability to habituate to day-to-day trauma reminders is believed to contribute to the persistence of PTSD. Studies of physiological responses to trauma-related stimuli in PTSD patients typically find differences from controls. PTSD patients reliably display an altered profile of persistent hyper-arousal, exaggerated startle responses [26,27], larger eye-blink, eye pupil, heart rate and slower skin conductance habituation [28]. These responses are less prominent for general negative stimuli.

While most studies of habituation to trauma-related stimuli in PTSD have measured physiological responses, at least one has used the emotional Stroop [29]. Habituation using the emotional Stroop is defined as diminished emotional interference effects (less RT slowing) combined with increased RT slowing for neutral words, or fatigue effects, over time [24,30]. The emotional Stroop has been used to assess habituation to relevant emotional words with healthy adults [31], individuals with panic disorder [24], individuals with elevated health anxiety [30] and veterans with PTSD [29]. McNally, Amir & Lipke (1996) [29] compared RTs by Vietnam combat veterans with and without PTSD over 4 mixed blocks of words (96 words each), in response to 4 word types: trauma, positive, neutral and color words. In a block by block comparison, they found that PTSD patients showed trauma-specific interference effects only on the first block, and then habituated to the content of the trauma words over time, becoming indistinguishable from controls by the end. However, McNally, Amir & Lipke’s 1996 study may have been limited in its ability to examine a habituation effect due to its small number of trauma-specific words (n=12) and the repetition of those stimuli.

The primary goal of our study was to expand on findings from the majority of emotional Stroop studies with PTSD patients, in which, compared to controls, PTSD patients exhibit significant interference (RT slowing) and increased errors on trauma-related words. But we also sought to examine some key questions about cognitive alterations in PTSD using the emotional Stroop. These questions included: 1) whether attentional biases are related to mere trauma exposure (with PTSD, or not), 2) whether emotional Stroop task performance would reflect the severity of PTSD symptoms, and 3) how a PTSD patient’s responses would change, over time, following exposure to trauma-related words.

We predicted that veterans with PTSD would show an enhanced emotional Stroop effect to Combat words, compared to control participants. Furthermore, we predicted that PTSD patients, but not trauma-exposed military controls, would show more interference from Combat words than from Negative words. We also predicted that emotional Stroop interference would correlate positively with scores on the PTSD checklist (PCL) and the Beck Depression Inventory (BDI) for all subjects. And finally, we expected veterans with PTSD to show diminished habituation to Combat words relative to controls.

## Methods

### Participants

Participants were recruited from clinics at the Department of Veterans Affairs Northern California Health Care System, through fliers placed in local military offices, and through internet postings. Thirty participants in each group participated in the study, including OEF/OIF war veterans with PTSD (PTs: 29 males; mean age in years=32.3, SD=7.9), military controls (MCs: 28 males; mean age in years=33.6, SD=8.3), and civilian controls (CCs: 28 males; mean age in years=32.2, SD=8.3). Exclusion criteria included any neurological or additional psychiatric disorders (i.e., schizophrenia, bipolar, epilepsy), having PTSD not due to OEF/OIF events (i.e., due to the Vietnam war, car accident, etc.), having a childhood TBI, or a moderate to severe TBI. Six participants who were initially enrolled were subsequently removed from the study (4 patients, 2 controls), when it was found they met exclusionary criteria (childhood TBI; nonmilitary PTSD; moderate TBI; other psychiatric disorder; not OEF/OIF). Two other participants did not complete the emotional Stroop task and were also subsequently removed from the study (2 patients). All subjects reported English as their first language. No subjects reported or displayed color vision deficits that affected performance. Demographic information is shown in Table 1.

**Table 1:** Demographic information and self-rating scores for patient and control groups

	**PTSD patients**	**Military controls**	**Civilian controls**
	**(n=30)**	**(n=30)**	**(n=30)**
Age (yrs)	32.3 ± 7.9 (24–51)	33.6 ± 8.3 (23–48)	32.2 ± 8.3 (20–49)
Education (yrs)	13.1 ± 1.5 (***) (8–16)	14.6 ± 1.7 (12–18)	14.8 ± 1.8 (12–20)
Handedness	27 R, 2 L, 1 ambi	26 R, 4 L	29 R, 1 ambi
Deployed (n)	30	19	---
Combat (n)	30	8	---
BDI	19.9 ± 9.3 (***)	5.5 ± 7.0	3.0 ± 3.16
PCL	57.7 ± 11.9 (***)	26.5 ± 10.9	26.0 ± 9.72
RTs (ms)	725.9 ± 17.04 (***)	604.4 ± 8.19	599.0 ± 9.45

*Note.* The mean ± standard deviation and range are given for age and education; the mean ± standard deviation for the BDI and PCL; and the mean ± standard error for reaction times. ***=significantly different from control groups at p<.001; R=right, L=left, ambi=ambidextrous; LOC=loss of consciousness (of 30 patients with mTBI, 21 had LOC, 5 did not, and 4 were not sure whether they had LOC); PCL=PTSD checklist; BDI=Beck Depression Inventory; RTs=reaction times.

#### Clinical Interview and diagnosis

The initial diagnosis of PTSD for VA-enrolled patients was made when a veteran sought help through the VA and received a semi-structured clinical interview by VA mental health providers using DSM-IV criteria. Mild TBI was diagnosed by a neurologist based on a semi-structured clinical interview and patient self-report of the following criteria from the VA/DoD Clinical Practice Guidelines – loss of consciousness 30 min or less or altered mental status (e.g., feeling dazed, disoriented, or confused), with post-traumatic amnesia less than 24 hrs [32]. Twenty-two of the 30 PTSD patients reported or were diagnosed with a mild traumatic brain injury (TBI), typically due to improvised explosive device (IED) blast exposure. Diagnosis of mTBI and PTSD in patients enrolled in our study was confirmed via a review of the VA’s Computerized Patient Record System (CPRS) and other available VA medical records to the fullest extent possible.

## Materials

### Emotional Stroop

The emotional Stroop task, a variant of the classic Stroop task, asks subjects to name the font color of emotional and neutral words, with instructions to ignore the meaning of the words. While the effects of the two Stroop tasks appear similar – a slowing in response times – they engage different interference mechanisms, with the classic Stroop creating a cognitive response conflict between an incongruent color and word (i.e., the word ‘RED’ in font color blue), and the emotional Stroop engaging an emotional response that interferes with the task demand of color naming.

The emotional Stroop stimuli used were colored words (red, blue, green, or yellow) shown one at a time in the center of a computer screen in 48 pt Times font, using all capital letters, on a black background at a distance of approximately 30 inches from the viewer. Colors did not repeat on consecutive words and were equally used throughout all trials.

The task included 5 blocks of words, with each block containing a single word category. The five categories of words were: 1) “Combat”: trauma-related words based in events of the OEF/OIF wars in Iraq and Afghanistan (i.e., *detainee, warlord, Falluja*); 2) “Matched-neutral”: words matched to combat words in number of letters and frequency (i.e., *detective, faculty, Jakarta*); 3) “Positive” (i.e., *proud, comedy, diamond*), “Negative” (i.e., *fraud, stupid, tragedy*) and “Neutral” (*sleep, poster, mixture*).

#### Combat and Matched-neutral words

We created the Combat word list from a search of mainstream media news stories, soldier blog entries, and other public sources describing unique and traumatic aspects of the OEF/OIF war experience. Typical OEF/OIF combat stressors included exposure to IED blasts and suicide bombers, seeing human remains, engaging in killing another person, experiencing violent deaths and injuries of fellow soldiers and friends, and being unable to stop violent situations [2]. Four types of Combat words were used: 1) Words associated with the OEF/OIF combat events (i.e., *insurgent*), 2) Place names (i.e., *Kirkuk*), 3) Military abbreviations (i.e., *IED*), and 4) General war trauma words (i.e., *gunmen*). Matched-neutral words were created by finding words neutral in valence to match Combat words on number of letters, syllables, word type and frequency (see Additional file 1).

#### Neutral, Negative and Positive words

Neutral, Negative and Positive words were matched on number of letters, number of syllables and frequency. Only high arousal Negative and Positive words were used and arousal and valence ratings for Neutral, Negative and Positive words were based on the Affective Norms for English Words [33]. ANOVAs were conducted to examine any word type differences. Mean valence ratings were as follows: Positive: 7.6 (SD=0.5, range=7.0–8.7), Negative: 2.6 (SD=0.6, range=1.3–3.9) and Neutral: 5.3 (SD=1.1, range=1.9–7.9). Arousal levels for both Positive (mean=5.8, SD=0.6) and Negative words (mean=5.8, SD=0.9) were higher than Neutral (mean=3.6, SD=0.4) (*p*<.0001). No significant differences between word categories were found using the Hyperspace Analogue to Language (HAL) frequency norms (*p*=0.69) from the online database of the English Lexicon Project (ELP) [34].

### Questionnaires: PTSD Checklist & Beck Depression Inventory

Following the emotional Stroop task, subjects were asked to complete the 17-item PTSD Checklist, Military or Civilian Version (PCL-M or PCL-C) to assess their level of PTSD symptoms during the past month. The PCL is a widely used 17-item self-report measure of the DSM-IV symptoms of PTSD [35]. Patients and military controls received the PCL-M (military), which asks about symptoms they have been bothered by in the past month due to “stressful military experiences”. The PCL-C (civilian) was given to civilian controls and asks about symptoms in response to “stressful experiences”. The PCL yields subscores for three different symptom clusters: re-experiencing, avoidance/numbing, and hyperarousal. All subjects were also given the Beck Depression Inventory (BDI; [36]), to assess levels of depression in the past few days. The BDI is a commonly used 21-item self-report screen for major depressive disorder (MDD) that has been validated with well-established psychometric properties [37].

### Procedure

Subjects signed informed consent forms approved by the Institutional Review Board of the Department of Veterans Affairs Northern California Health Care System and were paid $20/hr plus travel after completion of the session. All participants were instructed to name the color of a word shown on the computer screen by speaking into a voice-activated microphone as quickly and as accurately as possible. Participants started with 15 neutral word practice trials. Words were presented for 500 ms using Presentation software (Neurobehavioral Systems Inc., CA, USA), with a total trial time of 2000 ms and an inter-stimulus interval of 1500 ms. Each of the 5 blocks contained 84 words for a total of 420 unique words. Each block took approximately 3 minutes to complete and the study lasted between 15 and 20 minutes. Within blocks, words were presented in fixed pseudo-randomized order.

Because emotional stimuli can contaminate later non-emotional stimuli with carry-over slowing effects, the order of presentation of trials and blocks in an emotional Stroop study should attempt to counterbalance such effects [30,38]. We used a Latin Square design employed by McKenna and Sharma [31,39] to counterbalance order effects of different word types in a blocked design format across all participants. Blocks were counterbalanced using a balanced 5 × 5 Latin Square design [40,41] in which subjects received one of 10 possible block orders (5 block orders mirrored the other 5). Each of the 10 different Latin Square orders was repeated 3 times within each group (n=30).

Following the Stroop task each participant was debriefed on the purpose of the study and queried about their experience and whether they had any questions. The PCL and BDI questionnaires were administered on paper after the Stroop task and debriefing.

## Results

Only correct responses were included in results analyses (average percentage of error RTs removed: PTSD PTs=3.53%; Military controls=1.56%; Civilian controls=1.61%). Behavioral exclusion criteria included participants with more than 25% error rates [42] and no participants met that level. Trial reaction time data were trimmed to decrease variance such that RTs longer than 2 SDs above the subject’s block mean were removed (average removed: PTs=5.3%; MCs=5.4%; CCs=5.3%) [43], and RTs beyond 3000 ms or faster than 200 ms (i.e., coughs) [44], were removed (average removed: PTs=4.3%; MCs=3.6%; CCs=3.0%).

Reaction time and accuracy were each examined with a 3 × 5 Mixed Repeated Measures ANOVA, with Group (PTs, MCs, CCs) as the between-subjects factor and Word Valence (Combat, Matched-neutral, Neutral, Negative, Positive) as the within-subjects factor. When contrasts were not planned, a correction for multiple comparisons of *p*<.005 was used. No covariates were used in the analyses unless stated.

### Reaction times

#### Color-naming

**Figure 1:**
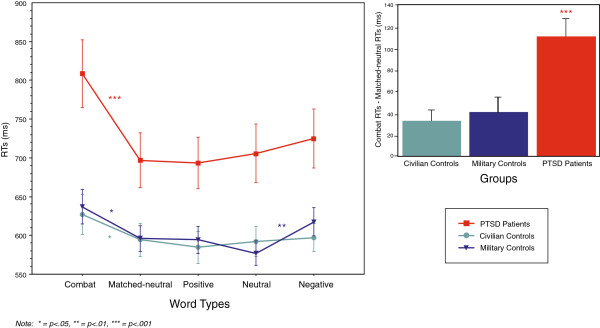
**Reaction times and Stroop interference scores.** Left: RTs for all blocks of word types. Error bars depict standard errors. Right: Stroop interference scores (Combat RTs minus Matched-neutral RTs). Error bars depict standard errors.

Reaction time results indicated a significant main effect of Group, *F*(2,87)=7.75, *p*=.0008, with overall RTs for PTs slower than either control group (Means: PTs=726 ms, MCs=604 ms, CCs=599 ms). A significant main effect was also shown for Valence, *F*(4,8)=26.16, *p*<.0001, with all groups slower on Combat words relative to Matched-neutral words (*p*<.02), confirming the emotional Stroop effect (see Figure 1). An interaction effect for Valence × Group, *F*(8,348)=3.87, *p*=.0002, indicated that group RTs differed depending on Word Type, with PTs showing greater slowing for Combat versus Matched-neutral than controls.

#### Combat and Matched-neutral

Within group planned paired t-test comparisons of Combat and Matched-neutral blocks showed that each group was slower on Combat words: PTs: *t*(1,29)=6.47, *p*<.0001; MCs *t*(1,29)=2.81, *p*=.009; and CCs *t*(1,29)=2.63, *p*=.01. A between-groups ANOVA analysis of RTs to Combat and Matched-neutral blocks showed a robust interaction of Valence x Group, *F*(2,87)=8.53, *p*=.0004, indicating that although all groups were slower on Combat words, PTs had greater slowing than either control group.

#### Neutral, Positive and Negative

Between group ANOVAs examining mean RTs on Negative versus Neutral and Positive versus Neutral blocks showed main effects of Group [Negative: *F*(2,87)=7.18, *p*=.001; Positive: *F*(2,87)=6.76, *p*=.002], with PTs significantly slower overall, but no significant group interactions (Negative: *p*=.11; Positive: *p*=.08). Within group planned paired t-test comparisons indicated that MCs were slower on Negative versus Neutral, *t*(1,29)=3.67, *p*=.001, and had a non-significant trend for being slower on Positive versus Neutral, *t*(1,29)=1.81, *p*=.08. CCs showed no significant differences on Negative versus Neutral, (*p*=.61), or Positive versus Neutral (*p*=.41) (see Table 2).

**Table 2:** Summary of word type comparisons by group

**Group**	**Comparison**	**RT difference**	***p***
PTSD patients	Combat vs Matched-neutral	112 ms	*p*<.0001
	Negative vs Neutral	19 ms	*p*=.19
	Positive vs Neutral	13 ms	*p*=.26
Military controls	Combat vs Matched-neutral	41 ms	*p*=.009
	Negative vs Neutral	41 ms	*p*=.001
	Positive vs Neutral	18 ms	*p*=.08
Civilian controls	Combat vs Matched-neutral	33 ms	*p*=.01
	Negative vs Neutral	5 ms	*p*=.61
	Positive vs Neutral	7 ms	*p*=.41

Thus, PTs did show a large interference effect on Combat words (112 ms; *p*<.0001) but not on Negative relative to Neutral (19 ms; *p*=.19). In contrast, MCs showed interference effects of a similar size on both Combat (41 ms; *p*=.009) and Negative (41 ms; *p*=.001) and CCs showed an interference effect by Combat words similar to MCs (33 ms; *p*=.01) but no other significant effects.

Although all groups were matched on age, they were not matched on education (Means in years: PTs=13.1, SD=1.5; MCs=14.6, SD=1.7; CCs=14.8, SD=1.8; *p*<.0003). Previous emotional Stroop studies of veterans with PTSD have also noted difficulty in matching groups of veterans on years of education (e.g., [11,13]). To test whether the slightly lower education in the PT group affected the findings of the study, we examined a subset of both control groups with lower education (n=32) to match with the PT group [mean education in years: PTs: 13.12; MCs: 13.44; CCs: 13.3 (*p*>.41)] and found that overall group RTs were still significantly different, *F*(2,59)=4.83, *p*=.01, and that the Group × Valence interaction still existed, *F*(8,236)=2.26, *p*=.02. The results were the same for the error analysis: while overall group accuracy was still significantly different, *F*(2,59)=6.12, *p*=.004, the Group × Valence interaction did not reach significance, *F*(8,236)=.886, *p*=.53. Only 8 MCs reported active combat, whereas all of the veterans with PTSD reported active combat. A between-groups ANOVA (MCs Deployed versus MCs Not Deployed) analysis of RTs did not find any overall group differences (*p*=.29) or Group × Valence interaction (*p*=.11). However, because the Latin Square order is not balanced in this type of analysis, the validity of such comparisons is difficult to determine.

#### Habituation

We analyzed habituation effects across the length of the Combat and Matched-neutral blocks (84 trials each) by comparing average RTs during each quarter of the blocks: “First quarter” (trials 1-21), “Second quarter” (trials 22-42), “Third quarter” (trials 43-63) and “Fourth quarter” (trials 64-84). The choice of quarters was based on the number of trials in the habituation analysis by Witthöft, et al. (2008) [30], which compared groups during the first and second halves of blocks (trials 1-20 and 21-40), and the emotional Stroop studies by McNally, Riemann & Kim (1990) [24] and McNally, Amir & Lipke (1996) [29], which analyzed 4 different word types, each occurring on 20 and 24 trials per mixed block (with each block being 100 and 96 trials in length), respectively.

**Figure 2:**
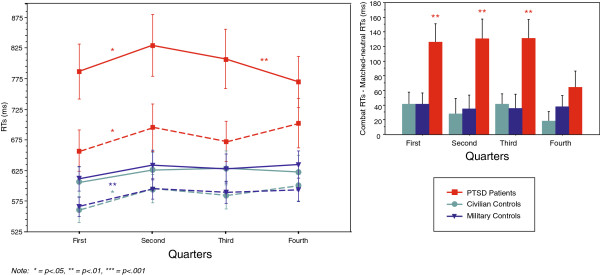
**Reaction times and Stroop interference scores.** Left: RTs for Combat (solid lines) and Matched-neutral (dashed lines) blocks across quarters. Error bars depict standard errors. Right: Stroop interference scores across quarters (Combat RTs minus Matched-neutral RTs). Error bars depict standard errors.

We analyzed RTs in a repeated measures 3 (Group) × 4 (Quarter) × 2 (Valence) ANOVA. Results showed an interaction effect of Valence × Group (*p*=.0008), no interaction of Quarter × Group (p=.54), and a trend for the 3-way interaction of Quarter × Valence × Group (*p*=.09) (See Figure 2). Planned t-test comparisons confirmed that PTs were slower on Combat than Matched-neutral words on all quarters, Q1: *t*(1,29)=5.1, *p*<.0001; Q2: *t*(1,29)=5.0, *p*<.0001; Q3: *t*(1,29)=5.1, *p*<.0001; Q4: *t*(1,29)=2.9, *p*=.007, while both control groups were slower only on quarter 1 (MCs: *t*(1,29)=2.8, *p*=.01; CCs: *t*(1,29)=2.7, *p*=.01), with intermittent slowing on other quarters (MCs: Q4, *p*=.02; CCs: Q3, *p*=.005).

In an analysis similar to McNally, Amir & Lipke (1996) [29], who found that trauma-related interference for veterans with PTSD was apparent only on the first of four blocks, we analyzed each quarter using a 2 (Group) × 2 (Valence) repeated measures ANOVA. Results indicated a significant Valence × Group interaction on quarters 1 – 3 (Q1: *F*(2,87)=6.48, *p*=.002; Q2: *F*(2,87)=6.7, *p*=.002; Q3: *F*(2,87)=7.24, *p*=.001), but not on quarter 4 (Q4: *F*(2,87)=1.81, *p*=.17). PTs showed a strong interference effect (over 120 ms) from Combat words during the first 3 quarters of the block, which decreased to 64 ms in the last quarter (See Figure 2), while control groups never showed more than 42 ms of interference slowing (See Table 3).

**Table 3:** Stroop interference across block quarters

	**PTSD patients**	**Military controls**	**Civilian controls**
First quarter	126.35 ms	41.66 ms	41.82 ms
Second quarter	131.23 ms	35.18 ms	27.99 ms
Third quarter	131.35 ms	35.89 ms	41.62 ms
Fourth quarter	64.36 ms	38.02 ms	18.73 ms

*Note*: Interference reaction times (RT) reflect Combat RT minus Matched-neutral RT.

These results suggest that although veterans with PTSD displayed exaggerated interference effects from trauma-related stimuli across the full length of the block, by the last quarter, the groups were no longer different. Thus, PTs tended to differ from controls for up to 63 trials, but appeared to habituate in the last quarter of the block.

### Accuracy

An ANOVA conducted for accuracy scores showed a significant main effect of Group *F*(2,87)=9.99, *p*=.0001, indicating that PTs were less accurate than control groups overall (average percent accuracy: PTs: 96.6; MCs: 98.4; CCs: 98.5). A main effect of Valence was also shown, *F*(4,8)=4.87, *p*=.0008, in which PTs were significantly less accurate than controls on four of the word types (*p*<.04) with a trend for Neutral as well (*p*>.07). A trend for a Group × Valence interaction was indicated (*p*=.11). Planned t-test comparisons of accuracy on Combat words showed that PTs were less accurate on Combat words relative to control groups, *t*(1,58)=-3.1, *p*<.003.

A speed-accuracy trade-off analysis using Spearman correlations indicated that CCs exchanged accuracy for speed on the Combat and Matched-neutral blocks: *r*(1,28)=.425, *p*=.02, while the trade-offs for PTs and MCs did not reach significance (*p*’s>.12). No other word types showed any significant speed-accuracy trade-off outcomes.

*Combat and Neutral Combat:* Planned paired t-test comparisons of Combat and Matched-neutral words within each group indicated a trend for PTs to be less accurate on Combat words, *t*(1,29)=1.97, *p*=.06, and no differences for control groups, (MCs:*p*=.54; CCs: *p*=.77).

*Neutral, Positive and Negative:* Planned paired t-test comparisons within each group for Neutral, Positive and Negative words revealed no accuracy differences (*p*>.14).

### Self-report questionnaires

PTSD patients reported higher PCL scores (58.1) than the military (27.1) or civilian (26.0) control groups, *F*(2,87)=51.2, *p*<.0001 (PTs vs MCs: *t*(1,58)=10.6, *p*<.0001; PTs vs CCs: *t*(1,58)=11.3, *p*<.0001) and higher BDI scores (20.4) than the military (6.3) or civilian (3.0) control groups, *F*(2,87)= 85.1, *p*<.0001 (PTs vs MCs: *t*(1,58)=6.78, *p*<.0001; PTs vs CCs: *t*(1,58)=-9.4, *p*<.0001). Bonferroni corrected comparisons between control groups indicated a non-significant trend for differences in depression on the BDI and no significant differences on the PCL (BDI, *p*=.07; PCL, *p*=.70).

### Correlations between experimental and self-report measures

Spearman correlations conducted between the PCL and BDI self-report measures and behavioral performance indicated interference from Combat words (larger RT difference for Combat minus Matched-neutral blocks) correlated positively with increased depression scores on the BDI (*rho*=.36; *p*=.0007), and PTSD symptoms on the PCL (*rho*=.33; *p*=.002). The same correlations with Negative word interference (Negative minus Neutral words) were not significant for the BDI (*rho*=.001; *p*=.99), or PCL (*rho*=.03; *p*=.75). Within the PCL, the PTSD symptom clusters of re-experiencing (*rho*=.38; *p*=.0005), hyper-arousal (*rho*=.33; *p*=.002), and avoidance/numbing (*rho*=.25; *p*=.02) also showed significant positive correlations.

## Discussion

We found that OEF/OIF veterans with PTSD had significantly more interference on trauma-related words relative to controls and displayed slower RTs and lower overall accuracy, replicating the findings of several previous studies using the emotional Stroop task with veterans with PTSD [7-13] Veterans with PTSD did not show interference on Negative or Positive words relative to Neutral, suggesting that their emotional Stroop response was specific to Combat words. Additionally, across groups, responses on the PCL and BDI questionnaires were positively correlated with interference slowing on Combat words, suggesting that increased severity of PTSD and depression symptoms were related to increased difficulty in inhibiting emotional interference on the task. Nonetheless, PTSD patients tended to display habituation to the Combat words, despite each word being novel and relatively specific to the OEF/OIF trauma environment. These findings support theories of a dysregulated fear response involving hypervigilance to trauma-related stimuli in PTSD patients, likely involving both an attentional bias and difficulty in disengaging from the stimuli.

The results of our study differed from most previous emotional Stroop studies of PTSD in that all groups – rather than only veterans with PTSD – showed significant interference from Combat words. This outcome may be due to the use of particularly salient and intense trauma-related words (i.e., *decapitate, abduct, severed, torture)* and that none of the words were repeated. Many studies of PTSD using the emotional Stroop use fewer and less unique words (i.e., *medevac, firefight)* and / or use words which are repeated [10,13,29,30]. Our design was used to assist in finding habituation effects, which could be diminished or confounded if words were repeated. It also delineated larger interference effects, as indicated by the fact that all groups showed interference effects to Combat words, and that despite this, PTs still had a significantly larger interference effect relative to controls.

In our study, the impact of trauma-related material on PTs appeared to eclipse the effects of Negative words, with Combat words generating much larger interference effects than Negative words. Veterans with PTSD sometimes reported feeling as though they were “awoken” by exposure to the Combat words, relative to the other blocks, and were perplexed by the experience in which they “could not take their eyes off the words”. Importantly, PTs showed no difference on Negative relative to Neutral words, an effect opposite to standard emotional Stroop results using a blocked design [16,39]. In contrast, the military control group (MCs) displayed the same slowing on Negative relative to Neutral as they did on Combat-related relative to Matched-neutral words (41 ms each). That the elevated emotional Stroop effect in PTs was specific to Combat words and did not generalize to other negative words, is supported by other studies that have found that the emotional Stroop task can index PTSD specifically [10,11,20,24,25]. Other factors, such as avoidance and numbing, may also be involved in these results. Foa and colleagues (1995) have suggested that the PTSD symptom of numbing – i.e., reduced interest, social withdrawal and emotional numbing – may be a compensatory mechanism in response to persistent hyperarousal when the distress of re-experiencing symptoms cannot be alleviated through avoidance [45]. A recent study of perceptual processing advantages for trauma-related information (but not for general threat pictures) in patients with PTSD and Acute Stress Disorder suggested that reduced awareness of stimuli considered safe and normal may play a role in the development and persistence of PTSD [46]. Reduced awareness, or numbing, could explain the lack of generalization of the emotional Stroop effect to negative words. Alternately, avoiding all trauma reminders could also result in the suppression of non-trauma stimuli as well, such as neutral or negative stimuli.

Another important question in our study was whether symptom severity would show a relationship to task performance on the emotional Stroop. We found that PCL and BDI questionnaire responses were positively correlated with the percent of interference shown on Combat words across groups, suggesting that increased severity of PTSD and depression symptoms were related to increased difficulty in inhibiting emotional interference on the task. Limitations in executive control processes [47] may contribute to the inability of PTSD patients to disengage from traumatic memories (re-experiencing) and to modulate emotional responses (hyperarousal). These in turn may lead to withdrawal from situations in which executive control is likely to fail (avoidance and numbing) [22].

Our study also examined habituation effects (RT decrease to Combat words) to assess the impact of trauma-related stimuli on veterans with PTSD over time. Hyperarousal and hypervigiliance are characteristics of PTSD which may contribute to deficits in habituation, resulting in difficulty adapting to repeated exposure to trauma-related stimuli. We found that veterans with PTSD showed consistently strong interference to Combat words (over 120 ms) for up to 63 trials. The only other study to use the emotional Stroop to examine habituation to trauma-related stimuli for veterans with and without PTSD over time [29], found group differences, but only in the first of four blocks, and only as a linear pattern of RT decreases over time. However, that study included just 12 different trauma-related words repeated 8 times using a mixed, rather than pure, block design. It is likely that methodological differences, as well as the novel, intense and trauma-specific nature of our word stimuli, led to the persistent and substantial interference effects seen in the current study. Importantly, however, despite the initial impact of the words, veterans with PTSD did tend to habituate and reach a color-naming response rate statistically indistinguishable from controls by the last quarter of the Combat block.

Although not a key question we sought to answer, we also found that veterans with PTSD showed significantly slower response times overall, relative to control groups. This finding is supported by other studies using the emotional Stroop to assess PTSD, which have found that generally, PTSD participants respond slower relative to healthy controls [12,13,48]. In contrast, results on a GoNoGo task administered to all subjects in our study indicated a striking lack of mean RT differences between PTs and controls, although the PTSD patients had significantly more RT variability and false alarm errors (see GoNoGo task in: [49]). Whether the overall slowing in our study could be due to the involvement of trauma-related emotional content, or some other factor, cannot be determined here and remains to be examined in future research.

It should be noted that any study investigating groups of war veterans may be limited by the availability of a completely comparable control group – that is, healthy veterans deployed to the war zone, engaged in active combat and exposed to trauma, but without PTSD or TBI, and available and motivated to participate in research. Within our group of 30 MCs, 19 were deployed to Iraq or Afghanistan and exposed to the OEF/OIF combat environment, without PTSD or TBI. In the case of the OEF/OIF wars, studies suggest that the factor of deployment alone (without combat or injury), compared with non-deployment, has been associated with neuropsychological compromise on basic cognitive tasks [50]. However, in our study there were no differences between MCs who were deployed (and potentially exposed to traumatic events) and those who were not.

Additionally, some believe that the PCL may overestimate PTSD prevalence, and that the civilian version of the PCL, the PCL-C, may not be anchored to a specific trauma, but may reflect negative emotionality rather than specific PTSD [51]. While our study cannot determine if the PCL-C reflects a specific trauma, we did not find differences between control groups on PCL scores (*p*=.9), despite the fact that our military control group (MC) received the PCL-M, while the civilian control group (CC) received the PCL-C. However, the PCL scores of both control groups did strongly differ from the patient group (*p*=.0001), which also received the PCL-M. If the PCL does overestimate PTSD prevalence, correlations to behavior may have been more difficult to find. Studies also indicate a high correlation between the PCL and the gold standard for diagnosis, the Clinician-Administered PTSD Scale (CAPS) [52].

Whether our findings of emotional Stroop differences in patients with PTSD due to combat trauma can be generalized to other types of trauma survivor groups remains a question for further analysis, and is a limitation of this study – trauma from combat is a relatively uncommon event and typically involves more men than women. Differences among types of PTSD trauma have been suggested, such as PTSD due to intentional assault being more severe and long lasting than PTSD from accidents or natural disasters. Additionally, since most of our PTSD patients reported or were diagnosed with a mild TBI (22 of 30), we were unable to conduct a meaningful comparison of patients with PTSD only versus PTSD + mild TBI. However, a separate study involving all of the same subjects [49] on a Go/NoGo task did compare PTSD only (n=10) and PTSD+ mild TBI (n=30) and found no group differences (*p*>88).

While we believe that our task improved on the design and methodology of existing emotional Stroop designs, some questions remain. For example, the group differences in interference effects from Negative words was unexpected and would benefit from further analysis: Would the results be the same if groups were tested only on Negative, Positive and Neutral words separately from trauma-related words? With careful design, possible interactions of trauma-related stimuli with other stimuli could be teased apart to strengthen the understanding of the best way to use the emotional Stroop to assess the impact of PTSD. Furthermore, a future pilot study could be conducted with a modified form of the emotional Stroop using only trauma-related and matched neutral word blocks to assess possible PTSD in veterans after their return from duty. Since interference effects in the emotional Stroop cannot easily be falsified [53], this simple behavioral task could provide a useful assessment tool.

## Conclusions

Despite some limitations, this study has shown that PTSD patients display a unique attentional bias for trauma-related words, but not general negative words, suggesting that the emotional Stroop task can index PTSD specifically. Our study also revealed that even with exposure to unique, high intensity, trauma-related words reflecting specific names and details from the trauma environment, veterans with PTSD display a tendency to habituate to these stimuli over time, supporting the use of interventions such as exposure therapy. Similarly, attentional retraining, as part of a trauma-focused intervention, or even as a preventive measure among those predisposed to PTSD, is also supported by our findings of the negative impact of attentional biases to trauma-related information in veterans with PTSD.

## Abbreviations

BDI: Beck Depression Inventory; CC: Civilian control group; MC: Military control group; OEF/OIF: Operation Enduring Freedom / Operation Iraqi Freedom; PCL: PTSD Checklist; PT: Patient group; PTSD: Posttraumatic stress disorder; TBI: Traumatic brain injury

## Competing interests

The authors declare that they have no competing interests.

## Authors’ contributions

VA: study design, data collection and analysis, manuscript preparation. NH, TJ & JL: data collection, manuscript preparation. DS: principal investigator, supervision, data analysis, manuscript preparation. All authors read and approved the final manuscript.

## Pre-publication history

The pre-publication history for this paper can be accessed here:

http://www.biomedcentral.com/1471-244X/13/86/prepub

## Supplementary Material

Additional file 1 **Word lists for each category of 84 words each.** Combat and Matched-neutral words include general combat words, city names, words unique to OEF/OIF, and abbreviations. Click here for file
