# Supplementary material for: Attentional bias for trauma-related words: exaggerated emotional Stroop effect in Afghanistan and Iraq war veterans with PTSD
Source: BMC Psychiatry. 2013 Mar 14;13:86. doi: 10.1186/1471-244X-13-86 (PMC3608167; doi:10.1186/1471-244X-13-86)
Supplement: Additional file 1 — Word lists for each category of 84 words each. Combat and Matched-neutral words include general combat words, city names, words unique to OEF/OIF, and abbreviations. [file 1471-244X-13-86-S1.doc]

APPENDIX

Word lists for each category of 84 words each. Combat and Matched-neutral words include general combat words, city names, words unique to OEF/OIF, and abbreviations.

| *Positive* | *Negative* | *Neutral* | *Combat* | *Matched-Neutral* |
| --- | --- | --- | --- | --- |
| hug | cut | boy | gun | van |
| joy | mad | hay | war | net |
| car | hit | shy | body | city |
| dog | lie | cat | bomb | week |
| eat | sin | bus | kill | move |
| sun | rat | air | tour | ride |
| toy | fat | bed | vest | vine |
| gift | hurt | door | medic | tenor |
| fame | dump | milk | shell | quote |
| cute | foul | dirt | abduct | obsess |
| grin | mold | slow | ambush | gossip |
| idea | rude | silk | Apache | Athena |
| snow | pity | seat | captor | caddie |
| song | jail | foot | combat | bottle |
| star | sick | safe | convoy | pastry |
| heal | lost | lamp | gunmen | sitter |
| baby | tomb | item | gunner | jurors |
| cake | burn | farm | kidnap | peruse |
| cozy | debt | bowl | martyr | dining |
| jolly | trash | bench | mortar | comets |
| proud | crime | rusty | patrol | skiing |
| puppy | slime | habit | sniper | tenant |
| honor | crude | bland | terror | permit |
| humor | slave | salad | weapon | expert |
| brave | roach | metal | airlift | roofing |
| lucky | alone | quart | captive | sunrise |
| music | scorn | plant | execute | examine |
| merry | loser | horse | explode | consume |
| loyal | blind | jelly | gunfire | biscuit |
| treat | snake | wagon | hostage | seniors |
| learn | dirty | slush | infidel | puritan |
| loved | filth | table | militia | antenna |
| silly | stink | sleep | missile | founder |
| truth | drown | chair | severed | resumed |
| child | thief | bored | suicide | faculty |
| cheer | fever | board | torture | thunder |
| glory | fraud | elbow | trigger | housing |
| champ | flood | tower | warfare | booklet |
| palace | malice | pencil | wounded | rounded |
| dollar | broken | solemn | amputate | renovate |
| dancer | wicked | poster | casualty | tapestry |
| strong | misery | violin | evacuate | unifying |
| travel | offend | square | militant | partisan |
| comedy | rotten | window | prisoner | observer |
| dinner | stupid | sphere | roadside | newsroom |
| savior | horror | gentle | shrapnel | trustees |
| mother | crisis | golfer | blindfold | blueprint |
| riches | hatred | moment | crossfire | staircase |
| bright | damage | butter | explosive | undefined |
| talent | rabies | engine | insurgent | condiment |
| joyful | poison | finger | checkpoint | paintbrush |
| trophy | insane | museum | concussion | complexion |
| honest | insult | basket | decapitate | redecorate |
| scholar | selfish | obesity | projectile | dishwasher |
| admired | tragedy | comfort | Anbar | Cairo |
| sunrise | trouble | packets | Basra | Paris |
| festive | useless | cottage | Kabul | Delhi |
| holiday | tornado | cabinet | Mosul | Milan |
| hopeful | garbage | staples | Bagram | Lisbon |
| fantasy | lawsuit | staying | Kirkuk | Moscow |
| devoted | delayed | symbols | Baghdad | Bristol |
| diamond | illness | cabbage | Falluja | Jakarta |
| victory | corrupt | shorter | Haditha | Nairobi |
| justice | outrage | retired | Kandahar | Damascus |
| liberty | destroy | arrange | caves | trunk |
| magical | divorce | history | behead | bestow |
| blossom | penalty | mixture | Mullah | Bishop |
| improve | poverty | relaxed | Muqtada | Juanita |
| adorable | disaster | bathroom | Taliban | Chianti |
| terrific | bankrupt | building | warlord | shipman |
| vacation | arrogant | umbrella | Zarqawi | Audubon |
| treasure | troubled | thorough | detainee | detective |
| inspired | contempt | windmill | firefight | fieldwork |
| laughter | confused | feathers | Kalashnikov | Appalachians |
| luscious | terrible | periodic | HV | UV |
| exercise | pressure | reserved | RPG | DVD |
| friendly | jealousy | performs | IED | DNA |
| champion | rejected | pamphlet | WMD | NBA |
| sapphire | ridicule | neighbor | APC | NFL |
| applause | sickness | corridor | CHU | PDA |
| ambition | starving | spinning | MOUT | RSVP |
| outdoors | dreadful | curtains | AK-47 | BLVD |
| pleasure | insecure | segments | KMTC | USPS |
| prestige | helpless | cylinder | VBIED | SCUBA |

*Note:* OEF/OIF = Operation Enduring Freedom / Operation Iraqi Freedom.
